# Neuroprosthetics in systems neuroscience and medicine

**DOI:** 10.1038/s41598-021-85134-4

**Published:** 2021-03-08

**Authors:** Kenji Kansaku

**Affiliations:** 1 Department of Physiology, Dokkyo Medical University School of Medicine, 880 Kitakobayashi, Mibu, Tochigi 321-0293 Japan; 2 Center for Neuroscience and Biomedical Engineering, The University of Electro-Communications, Tokyo, Japan

**Keywords:** Brain-machine interface, Translational research

## Abstract

The impaired brain is often difficult to restore, owing to our limited knowledge of the complex nervous system. Accumulating knowledge in systems neuroscience, combined with the development of innovative technologies, may enable brain restoration in patients with nervous system disorders that are currently untreatable. The *Neuroprosthetics in Systems Neuroscience and Medicine Collection* provides a platform for interdisciplinary research in neuroprosthetics.

The philosopher Stiegler suggested that human beings are essentially prosthetic creatures due to our propensity to create tools, such as glasses and smartphones^1^. In the same vein, patients are usually eager to restore lost physiological functions of body parts using prosthetic devices. Neuroprosthetic devices can substitute for motor, sensory, or cognitive functions that have been impaired as a result of nervous system disorders. The most successful neuroprosthetic devices developed to date are cochlear implants for patients with hearing impairment, and prosthetic devices for amputees. Both classes of device are located on the edge of the nervous system’s sensorimotor processing, so replacement using these devices was relatively simple, although the functions of certain consumer products have been somewhat limited.

Systems neuroscience is a subdiscipline of neuroscience that studies brain function at the system level. The nervous system is made up of an enormous number of neuronal cells. Therefore, its complexity is a barrier to gaining a deep understanding of the nervous system and for creating high functioning neuroprosthetic devices. However, through focusing instead on neural circuits, systems neuroscience has provided a number of recent insights, which have furthered our understanding of the brain. Such advances in systems neuroscience combined with the development of innovative technologies may enable brain restoration in a greater number and diversity of patients with nervous system disorders. This *Collection* provides a platform for interdisciplinary research in neuroprosthetics (Fig. 1). It consists of three subcategories:

1. Medical applications of sensorimotor neuroprosthetics.
2. Systems neuroscience and neuroprosthetics.
3. Next-generation technologies for neuroprosthetics.

**Figure 1:**
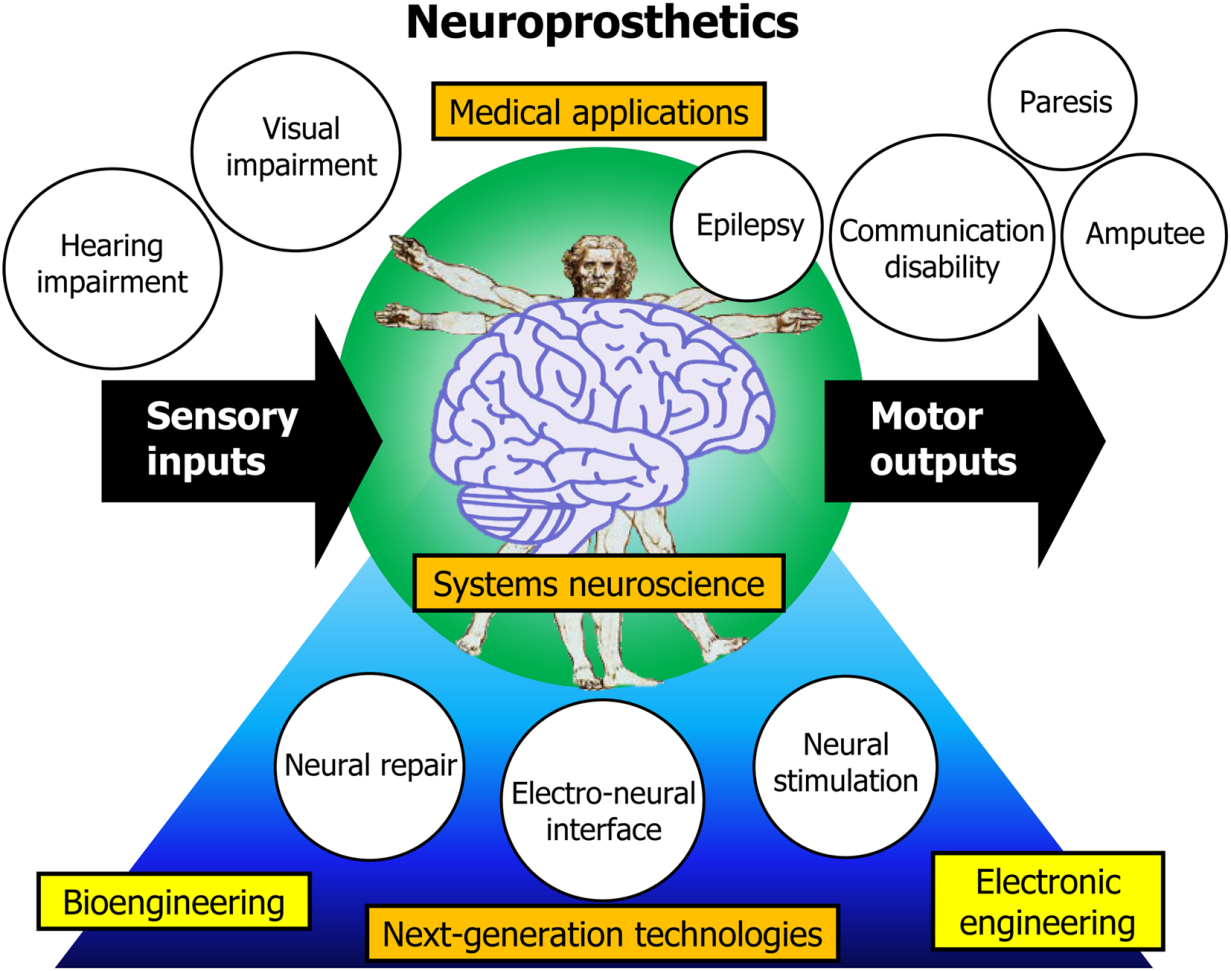
Schematic figure showing the components of the *Neuroprosthetics in Systems Neuroscience and Medicine Collection*.

The first subcategory, *Medical Applications of Sensorimotor Neuroprosthetics*, involves the accumulation of clinical studies in patients to investigate future neuroprosthetics. As described above, the most successful neuroprosthetic devices developed to date are cochlear implants for patients with hearing impairment and prosthetic devices for amputees. Therefore, there has been a great deal of research regarding these devices, located on the edge of the sensorimotor processes. However, most of the research discussed here is not specifically focused on simple sensory input or motor output, but rather is related to interactions occurring during sensorimotor processing. For example, Micera and colleagues developed tactile sensors, using morphological neural computation approaches, to deliver intraneural peripheral stimulation, and reported that amputee participants could successfully discriminate naturalistic textures^2^.

Neuroprosthetic devices have also been developed for paretic patients, aimed not only at restoring but also to rehabilitating motor function^3^. Researchers record signals directly from the brain and connect them to effectors using technology referred to as a brain–machine interface^4^. To utilize such technologies for neuroprosthetic devices, biosignals have been recorded either noninvasively^5^ or invasively^6^. The primary motor cortex has been the main area from which motor-related information is extracted, but current research discussed in the *Collection* involved the recording of signals from the middle frontal gyrus^7^. Thus, more complex sensorimotor information from the brain could be extracted to restore motor function. It should be noted that patients with loss of voluntary movements also lose their means of communication, because communication is closely linked to movements of body parts. Pioneering work using brain–machine interface technology for such patients was reported by Birbaumer and colleagues^8^, and studies along this line have been performed continuously over the last 20 years^9–11^.

Other medical procedures that can be categorized as neuroprosthetics are vagus nerve stimulation or responsive neurostimulation for medically refractory epilepsy, and deep brain stimulation for movement disorders. Investigations to improve medical practices in this area are currently in progress^12^.

The second subcategory, *Systems Neuroscience and Neuroprosthetics*, gathers research performed in able-bodied participants or in vivo animal studies to investigate complex brain functions at the system level. For example, Fletcher and colleagues asked normal-hearing participants to use their new device to provide missing pitch perception through haptic stimulation on the forearm, and these participants showed enhanced pitch discrimination when hearing cochlear implant simulated audio^13^. This study was planned because it has been noted that users of traditional cochlear implants have severely impaired pitch perception.

Clinical experiences also motivate researchers to perform in vivo animal experiments. For example, as it is known that vagus nerve stimulation sometimes induces neuropsychiatric effects, Takahashi and colleagues performed studies in rats and found that vagus nerve stimulation induced layer-specific modulation of evoked responses in the sensory cortex^14^. Newly acquired knowledge based on systems neuroscience research performed in able-bodied participants, or using in vivo animal models, will aid the future development of neuroprosthetics with suitable functions for the treatment of nervous system disorders.

The third subcategory, *Next-generation Technologies for Neuroprosthetics*, is related to research into the application of advanced engineering technologies or informatics methodologies, such as in vitro bioengineering or electronic engineering. In vitro bioengineering studies have often used human or animal cells. For instance, Kubinová and colleagues used human umbilical cord cells to investigate extracellular matrix hydrogel as a scaffold for neural tissue repair, and showed that crosslinking with genipin improved the biostability of hydrogels^15^.

One topic of electronic engineering research is the development of biocompatible electrodes for application in either a noninvasive^16^ or an invasive^17^ manner. In this *Collection*, Flores and colleagues developed an electrode array with a honeycomb configuration and demonstrated the migration of retinal cells into voids in the subretinal space. This new technique may allow the development of cell-scale pixels in subretinal prostheses^18^. Interfacing technology between organisms and artificial objects is key to the development of practical neuroprosthetic devices.

The brain system is extremely complex and, therefore, the fact that the development of neuroprosthetics trials has often been based on a single discipline may have been a limitation. This *Collection* is aimed at providing a platform for interdisciplinary approaches to create new neuroprosthetics, with the aim of contributing to future medical practice for the treatment of patients with nervous system disorders.
